# A Letter is a Letter and its Co-Occurrences: Cracking the Emergence of Position-Invariance Processing

**DOI:** 10.3758/s13423-023-02265-7

**Published:** 2023-05-05

**Authors:** Maria Fernández-López, Manuel Perea

**Affiliations:** 1 Department of Methodology of Behavioral Sciences and ERI-Lectura, Univesity of València, Facultat de Psicologia i Logopèdia, Blasco Ibáñez Avenue 21, 46010 València, Spain; 2 Nebrija University, Madrid, Spain

**Keywords:** Orthographic processing, Orthographic regularities, Letter position coding, Artificial script

## Abstract

Visual word recognition requires encoding letter identities and positions (orthographic processing). The present study focuses on the emergence of the mechanism responsible for encoding letter order in a word: position invariance. Reading experience leads to developing a flexible mechanism that encodes the information of the position of letters, explaining why jugde and judge are easily confused. Critically, orthographic regularities (e.g., frequent letter co-occurrences) modulate letter position encoding: the pseudoword mohter is extremely similar to mother because, in middle positions, the bigram TH is much more frequent than HT. Here, we tested whether position invariance emerges rapidly after the exposition to orthographic regularities—bigrams—in a novel script. To that end, we designed a study with two phases. In Phase 1, following Chetail (2017; Experiment 1b, *Cognition*, *163*, 103–120), individuals were first exposed to a flow of artificial words for a few minutes, with four bigrams occurring frequently. Afterward, participants judged the strings with trained bigrams as more wordlike (i.e., readers quickly picked up subtle new orthographic regularities) than the strings with untrained bigrams, replicating Chetail (2017). In Phase 2, participants performed a same–different matching task in which they had to decide whether pairs of five-letter strings were the same or not. The critical comparison was between pairs with a transposition of letters in a frequent (trained) versus infrequent (untrained) bigram. Results showed that participants were more prone to make errors with frequent bigrams than with infrequent bigrams with a letter transposition. These findings reveal that position invariance emerges rapidly, after continuous exposure to orthographic regularities.

## Introduction

 If we consider reading using architectural terms, its building blocks are words, which, in alphabetical languages, are made up of letters. There is broad consensus that the identification of a written word is mediated by a process in which, from sensory input, the word recognition system encodes the abstract identities of letters in a specific order, thus allowing us to discriminate hiss from kiss and dog from god. This bridge between the sensory input and the word level has been termed orthographic processing (i.e., the encoding of letter identities and positions; see Grainger, 2018).

 Previous research has shown that the way the human brain encodes the positions of letters in alphabetic languages is fairly flexible (see Massol & Grainger, 2022). For instance, when participants are asked whether two successive strings of letters are the same or different, they respond “different” more slowly (and with less accuracy) to transposed-letter pairs (e.g., CFLZ–CLFZ) than to control, replaced-letter pairs (e.g., CFLZ–CDVZ). Importantly, this effect is also sizable for strings of numbers (7586–7856) and symbols (£§?@–£?§@), which suggests that there is some general uncertainty in assigning the position to objects (e.g., letters) in a string. Thus, the letters F and L in CFLZ would activate their own and neighboring positions, explaining the difficulty of responding “no” to CFLZ–CLFZ in same–different tasks (perceptual-based models; e.g., overlap model, Gomez et al., 2008; spatial coding model, Davis, 2010; Bayesian reader, Norris & Kinoshita, 2012). Critically, the magnitude of transposition effects is larger for strings of letters than for strings of other visual objects (Duñabeitia et al., 2012; see also Fernández-López et al., 2021; Ktori et al., 2019; Massol et al., 2013; Massol & Grainger, 2022). The most suitable explanation for this dissociation is that an orthographic mechanism operates on top of perceptual uncertainty (see Grainger, 2018; Marcet et al.,^2019^)—this proposal goes back to Estes (1975).

 The present paper focuses on the emergence of the orthographic mechanism responsible for encoding the “relative positions of a set of object identities” (i.e., position invariance, which is the encoding of the order of visual objects [letters] in a string composed of several objects [a word]). Reading experience leads to the development of a flexible mechanism that, to host a unique word identity, encodes the information of the position of the letters with less precision (see Grainger & van Heuven, 2004; Whitney, 2001). This mechanism is based on a combination of ordered pairs of letter co-occurrences (called “open bigrams”). According to these models, the word mother would be composed of the open bigrams MO-MT-MH-ME-MR-OT-OH-OE-OR-TH-TE-TR-HE-HR-ER, where MO would refer to “M to the left of O”. If two contiguous letters from mother are switched—as in mohter, 93% of the bigrams remain unchanged, thus explaining why mohter is confusable with mother—or CLFZ with CFLZ.

Notably, letter order coding cannot be reduced to an open-bigram mechanism that encodes pairs of letters in a given order but does not distinguish whether the two letters are contiguous. In their dual-route model, Grainger and Ziegler (2011) proposed that, when encoding frequent complex graphemes such as th in mother, readers know that H *immediately* follows T, not just that H is somewhere after T (see also Goswami & Ziegler, 2006). Thus, besides a flexible orthographic route where open bigrams help identify a word, there is a more precise coding route based on chunking recurrent co-occurring letter combinations (e.g., TH, CH, or SH). In this scenario, the pseudoword mohter would be easily confused with mother not only because of perceptual uncertainty (common to all visual objects) or sharing many open bigrams, but also because HT is a nonfrequent bigram that could be misperceived with the frequent chunk TH. The logic is that orthographic knowledge would affect the perception of letter strings, so that the information from the visual input could be distorted to perceive the stimulus as regular (i.e., the “most probable interpretation of the graphemic input”; see Rumelhart, 1985, p. 732).

The knowledge of the letter sequences that normally occur at different word positions is acquired via repeated exposure to printed words. To reduce the amount of information to be processed, reading experience and print exposure lead to the implicit learning of orthographic regularities (e.g., facts about the distribution of letter co-occurrences). Indeed, orthographic regularities in the form of two-letter co-occurrences modulate the assignment of letter position in letter strings and words. In a perceptual identification task with briefly presented stimuli, Rumelhart (1985) reported that participants tended to commit transposition errors for letter strings containing illegal bigrams, such as praikc—note that KC is not legal at the end of words in English. Participants often reported praick instead, which includes the frequent complex bigram CK. Similarly, Frankish and Turner (2007) observed very high error rates for transposed-letter pseudowords like sotrm (base word: storm) in a lexical decision task, despite containing illegal bigrams—one might have thought that it would be easy to respond “nonword” based on this illegality (see also Frankish & Barnes, 2008; Perea & Carreiras, 2008, for converging evidence of greater transposed-letter effects for stimuli containing illegal transpositions using masked priming).

Prior research has shown that readers quickly embrace sublexical regularities. This is consistent with the idea that to simplify the inherent complexity of reading, orthographic processing relies on the regularities of the written system and capitalizes on statistical cues like bigram frequency ( Cassar & Treiman, 1997; Chetail, 2017; Lelonkiewicz et al., 2020; Mano & Kloos, 2018). Crucially, this ability emerges very rapidly throughout the exposure to print. A paradigmatic case is a study conducted by Pacton et al. (2001). They found that French readers were able, from very early in their development (i.e., 6 years old), to discriminate a word-like stimulus from a non-word-like stimulus based on their implicit learned knowledge of orthographic regularities. For instance, when comparing ommera vs. ovvera, readers preferred ommera because v is never doubled in French. That is, the participants relied their decision on the frequency of the bigrams mm vs. vv (see also Doignon-Camus & Zagar, 2014; O’Brien, 2014). While the above findings are very informative, they suffer from an inherent drawback. The effects of orthographic regularities such as bigram frequency cannot be easily disentangled from other relevant factors that influence visual word recognition: pronounceability, familiarity, or orthographic neighborhood (Chetail, 2017). Keep in mind that the frequency of the bigrams in a given language cannot be manipulated but must be selected; thus, the design cannot be genuinely experimental.

A practical strategy to overcome the above limitation is to use artificial scripts. This was the approach that Chetail (2017) followed with adult readers, testing what type of orthographic regularities emerges quickly with the exposition of print material in a novel script. Specifically, participants were first exposed to a flow of five-letter words in an unfamiliar script—Phoenician alphabet—for approximately 9 minutes. There were four trained bigrams, so each word was made up of one of these bigrams, always in the same position (see Table 1). Thereupon, in a wordlikeness task, participants were more likely to judge a new string as similar to the strings learned in the exposure phase if the string contained one of the trained bigrams in its position (i.e., a frequent bigram). A few minutes of exposition were enough to develop considerable sensitivity to bigram frequency. Chetail (2017) successfully replicated these findings in a second experiment in which participants learned 32 artificial words with a phonological form (i.e., the print-to-sound correspondences) before performing the task. Chetail (2017) concluded: “The statistical learning operating on the stream of artificial words made of a sequence of new shapes may be already oriented towards orthographic processing” (p. 118; see Vidal et al., 2021, for an alternative explanation). This study, however, did not test whether participants were prone to position-invariant encoding after learning the new orthography.

**Table 1:**
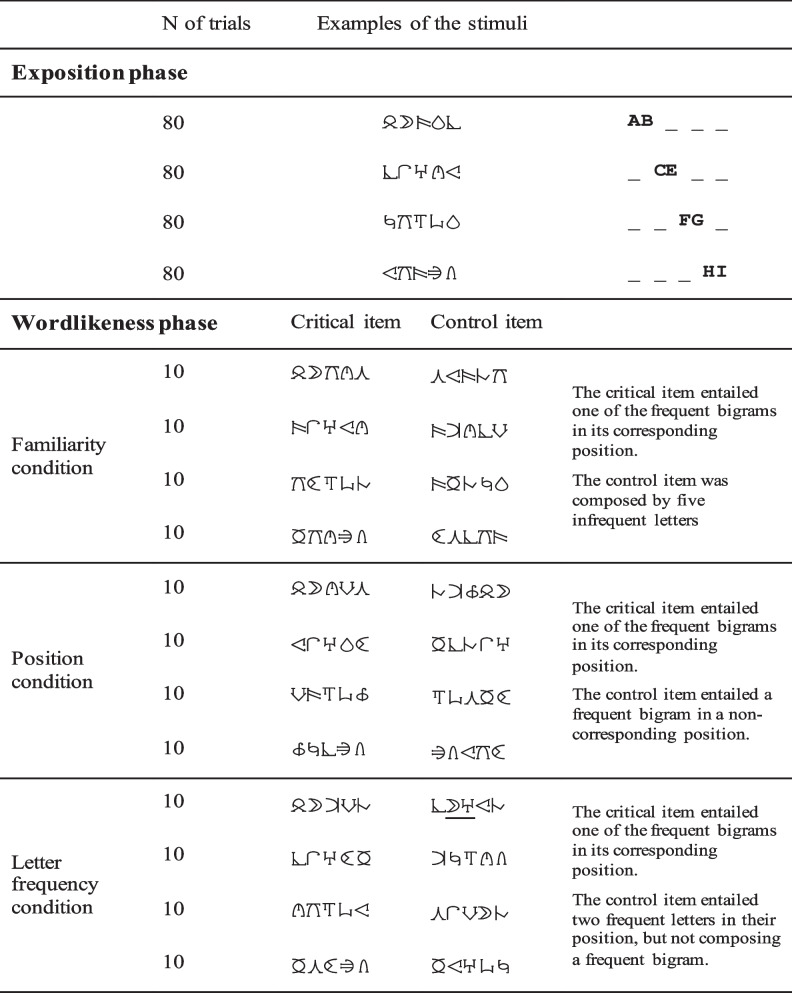
Reproduction of materials used in Phase 1: Replication of Chetail’s (2017) protocol

In Experiment 1b, Chetail (2017) used Phoenician alphabet—the database of artificial characters, BACS, was published in 2017. Boldface is ours, to emphasize the frequent bigram

The current experiment scrutinizes the emergence of position invariance using a protocol parallel to Chetail’s (2017) Experiment 1b. A recent study (Fernández-López et al., 2021, Experiment 1) failed to obtain evidence of position-invariant processing in an experiment in which participants learned to fluently read and write in a new script across six training sessions. The authors conducted, both before and after the training, a same–different matching task where the different trials were created by transposing or replacing two adjacent letters. Results showed a similar pattern of letter transposition effects posttraining and pretraining. The authors concluded that orthographic processing, at least in the form of position-invariant processing, does not emerge rapidly after learning a new script. However, they did not directly manipulate the sublexical properties of the stimuli. Here, we directly tested whether bigrams could be a key sublexical property helping the emergence of position invariance (see Grainger, 2018).

The present study was composed of two phases. In Phase 1, we reproduced Chetail’s (Chetail, 2017, Experiment 1b) procedure—we replicated the main findings. The novelty of our work relies on the addition of Phase 2, including a same–different matching task with transposed-letter pairs. In this task, the probe was a five-letter string presented for 300 ms and was immediately followed, one line below, by a target that could be the same or different. The critical comparison was the following: pairs in which the target was created by transposing a trained bigram (e.g., **AB**; probe: **AB**UVX, target: **BA**UVX) versus pairs in which the target was created by transposing an untrained bigram (e.g., **ZX**; probe: **ZXFG**U, target: **XZFG**U).^1^ As is common in this paradigm, we also included replacement-letter pairs: We replaced a frequent bigram with two frequent letters (e.g., probe: **AB**UVX, target: **EF**UVX) or replaced an infrequent bigram with two infrequent letters (e.g., probe: **XVFG**U, target: **MNFG**U)—this comparison explored whether the replacement of a frequent bigram makes the pair less perceptually similar than the replacement of an infrequent bigram.

Thus, the present experiment examined whether acquiring orthographic regularities (i.e., bigram frequency) modulates how letter order is encoded in a new orthography. Two outcomes are possible: If position invariance emerges rapidly from the representations created by the trained bigrams in the new script (e.g., **AB**), the sequence BA (BAUVX) could be confusable with **AB** (**AB**UVX) because of (1) perceptual uncertainty and (2) **AB** (but not BA) having a precise mental representation. Instead, ZX (ZX**FG**U) would produce some activation on XZ (XZ**FG**U) based on perceptual uncertainty alone. Therefore, it would be more difficult to respond “different” (i.e., more errors, longer response times) for those pairs involving the transposition of a frequent bigram like **AB** (position uncertainty + bigram activation) than an infrequent bigram like XZ (position uncertainty). This outcome would provide the first demonstration of the rapid emergence of position invariance. Alternatively, the trained bigrams in the exposure phase may not yet have formed stable representations to allow position invariance. If so, pairs with a letter transposition in trained or untrained bigrams would produce the same results (i.e., transposition errors would be based on perceptual uncertainty alone). This latter outcome would suggest that the emergence of position invariance requires more extensive reading experience.

## Method

### Participants

Thirty-six undergraduate students from the University of València participated in the experiment. This sample size, the same asChetail (2017, Experiment 1b), allowed us to have 1,440 observations per condition for the critical comparison of transposition of trained versus untrained bigrams, which is in line with Brysbaert and Stevens’s (Brysbaert & Stevens, 2018) recommendations. We also calculated Bayes factors (BFs) to obtain a measure of the evidence for or against the effect—of note, we found conclusive evidence in favor of a difference between the transposition of frequent versus infrequent bigrams in the accuracy data (BF = 112.16).^2^ All participants were native speakers of Spanish with normal or corrected vision and no history of reading or hearing disorders. They signed an informed consent form before participating in the experiment, and the study was approved by the Experimental Research Ethics Committee of the University of València. Participants received a small monetary compensation.

### Materials

We used 21 letters from the BACS font to devise the stimuli (BACS1 and BACS2 serif font; Vidal et al., 2017)—note that these characters were matched to the Roman script in complexity, number of strokes, junctions, and terminations (see Table 1 for an illustrative example of the stimuli).

For the *exposure phase*, we created 320 items of five characters, each including a critical bigram. Eight characters were used to devise the four frequent bigrams (frequent bigrams:  ,  ,  ,  are represented in the body of the manuscript as **AB**___, _**CE**__, __**FG**_, ___**HI**; the infrequent bigrams were formed by the letters  ). Each frequent bigram occurred in a specific position. In this manner, we created 80 items with the critical bigram in Positions 1 and 2 (**AB**KOR; bold is ours to facilitate the identification of the frequent bigram); 80 in Positions 2 and 3 (R**CE**TN); 80 in Positions 3 and 4 (ZX**FG**O), and 80 in Positions 4 and 5 (UFK**HI**). We also created 16 five-letter strings with Roman characters to act as fillers. Importantly, each stimulus contained nonrepeated characters. We created four different lists to counterbalance the position of the critical bigrams.

For the *wordlikeness phase*, as in Chetail (2017), we created 120 pairs of new stimuli (40 items per condition). For the familiarity condition, the critical item entailed one of the frequent bigrams in its corresponding position (based on the exposure phase; 10 items per position). The control item was composed by five random characters of the pool infrequent letters (e.g., UFK**HI** vs. BNPAO). In the position condition, the critical items were paired with control items that included the same critical bigram, but in a different position than in the trained items (i.e., UFK**HI** vs. **HI**BNP). Finally, in the letter frequency condition, the critical items were paired with control items that entailed two frequent letters in their frequent position, but they did not compose a critical bigram (UFK**HI** vs. MO**EG**B; see Table 1 for an illustration of the materials created for each condition). As in the exposure phase, we created four different lists to counterbalance the position of the critical bigrams. We also created six five-character string pairs to act as practice trials.

For the *same–different matching task*, we created 320 five-character string pairs (probe and target) in BACS font. All character strings were composed of nonrepeated letters. There were 160 same pairs and 160 different pairs. For the same pairs, 80 contained a frequent bigram in its standard position (**AB**UVX—**AB**UVX), and 80 were composed of infrequent letters (OVNKM—OVNKM). For the different pairs, 80 were created by transposing two letters, and 80 were created by replacing two letters—all of them contained a frequent bigram in its standard position. The transposed-letter pairs were created by transposing two adjacent letters in the target, that could be frequent (**AB**UVX—**BA**UVX; 40 pairs of items) or infrequent (**XZ**FGU—**ZX**FGU; 40 pairs of items). The replaced-letter pairs were created by replacing two adjacent letters in the target, which could be frequent (**AB**UVX—**CG**UVX; the replacement letters were other frequent letters not constituting a frequent bigram [**C** from CE and **G** FG]; 40 pairs of items) or infrequent (XV**FG**U—MN**FG**U 40 pairs of items). Each manipulation occurred in four different positions (1^st^-2^nd^, 2^nd^-3^r^, 3^rd^-4^th^, and 4^th^-5^th^)—there were 10 pairs of items per position. To counterbalance the position of the frequent bigrams, we created four lists following a Latin square. For the practice phase, we created eight additional five-character string pairs.

### Procedure

Each participant performed the tasks of familiarization, exposure, wordlikeness (Phase 1), and same–different (Phase 2). The tasks of Phase 1 paralleled those employed Chetail (2017). Participants were tested either individually or in groups of two in a quiet room. DMDX software (Forster & Forster, 2003) was used to display the sequence of stimuli and to register the timing/accuracy of the responses. All stimuli were presented in a monospaced font (15-pt BACS for the artificial strings; 15-pt Courier New for the Roman letters) in black on a white background. Response times were measured from target onset until the participant’s response. The whole session lasted about 30–40 min.

The *familiarization task* consisted of introducing the 21 new letters, presenting them one by one in a computer screen. Participants were told to hand-copy the characters on a sheet of paper, without a time deadline. Immediately after, all the new letters were presented and participants could look at them as long as necessary.

In the *exposure phase*, the 320 artificial character strings were presented individually in the center of the screen. Display duration and inter-stimuli interval were 1,200 and 500 ms, respectively. Participants were asked to carefully look at the stream of stimuli. To ensure that participants focused on the letter strings, 5% of trials were fillers composed of five letters in Roman script (e.g., MNRLT). The participants were asked to respond to fillers by pressing the space bar.

In each trial of the *wordlikeness task*, a pair of stimuli was presented (critical and control items) on the screen. The critical item was on the left part of the screen in 50% of the trials and on the right part in the other trials. Participants were asked to decide which stimulus was more similar to those presented in the exposure phase by pressing the corresponding key on the keyboard. They were asked to decide as soon as possible, although there was no time boundary.

In the *same–different matching task*, participants were told that they would be presented with two strings of characters and that they would have to decide if they were the same or not by pressing the “yes” and “no” keys. Participants were instructed to make this decision as quickly and accurately as possible. A fixation point (*) was displayed for 500 ms in the center of a computer screen on each trial. Next, the fixation point was replaced by a probe, which was presented for 300 ms and positioned 3 mm above the center of the screen. Then, the target item appeared one line 3 mm below the center of the screen. The target remained on the screen until the response or 2,000 ms had passed—in this latter case, the trial was categorized as an error response.

## Results

The analyses and results of Phase 1 are available in Appendix A—they essentially replicated Chetail’s (2017) findings: Participants were more likely to identify the foil containing previously learned bigrams as “wordlike” than that containing unfamiliar bigrams. For the inferential analyses of Phase 2 (same–different task), the dependent variables were the correct RT and accuracy. Very fast responses (<250 ms: nine responses) were omitted from the analyses of the correct RTs. Following our research goal, we tested the effect of the bigram frequency on transposed-letter processing—the analyses of “same” pairs and replacement-letter pairs are available in Appendix B. We fitted the data with Bayesian linear mixed-effects models using brms (Bürkner, 2021) in R (R Core Team, 2021). The fixed effect was bigram frequency (frequent vs. infrequent) with the maximal random effect structure model for subjects and items. We used the Gaussian distribution to model the latency data (−1,000/RT) and the Bernoulli distribution to model the accuracy data (1 = correct, 0 = incorrect). For each model, we employed 10,000 iterations in each of the four chains (2,000 warmup + 8,000 sampling). The chains converged successfully (all R̂s = 1.00). The output indicates the estimate of each effect (the mean of the posterior distribution), together with its standard error and 95% credible interval (95% CrI). We inferred evidence of an effect when its 95% CrI did not include zero—this was complemented by its BF. All the analyses are available online (https://osf.io/nst8w/?view_only=99edab01958a4d7cb8e8dc0c44d001ea).

The models showed that accuracy was noticeably lower when the letter transposition involved a frequent than an infrequent bigram (54.7 vs. 62.0, respectively; *b* = 0.33, *SE* = 0.09, 95% CrI [0.15, 0.50], BF = 112.16). The effect in the RTs had the same direction but was marginal (657 vs. 648 ms for the transpositions with frequent vs. infrequent bigrams),b = −0.03, *SE* = 0.02, 95% CrI [−0.06, 0.01], BF = 0.14 (see Table 2).

**Table 2:** Parameter estimates in accuracy

	Estimate	*SE*	95% credible interval
Intercept	0.19	0.06	[0.08, 0.30]
**Bigram frequency**	**0.31**	**0.08**	**[0.15, 0.47]**
**Position (linear)**	**−0.46**	**0.11**	**[−0.68, −0.25]**
Position (quadratic)	0.15	0.11	[−0.06, 0.35]
Position (cubic)	−0.05	0.11	[−0.26, 0.16]
Position (linear) × Bigram freq.	−0.12	0.15	[−0.42, 0.18]
Position (quadratic) × Bigram freq.	−0.09	0.15	[−0.39, 0.22]
Position (cubic) × Bigram freq.	0.11	0.15	[−0.19, 0.41]

Those effects with 95% credible intervals beyond zero are in boldface

**Fig. 1:**
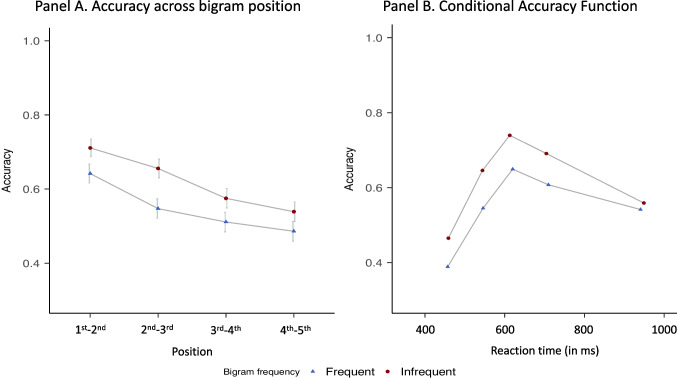
Panel **A:** Accuracy across the position of the transposed letters for frequent and infrequent bigrams. Panel **B:** Conditional accuracy functions. The points represent the accuracy and average RT of the responses within equal sized bins (20% of responses per bin)

To scrutinize the effect of bigram frequency on transposed-letter pairs, we conducted two additional analyses. In the first analysis, we added the position of the transposed letters (1^st^-2^nd^, 2^nd^-3^rd^, 3^rd^-4^th^, 4^th^-5^th^) as a polynomial factor in the design when analyzing accuracy. Results replicated the effect of frequency (*b* = 0.31, *SE * = 0.08, 95% CrI [0.15, 0.47]) and also revealed a linear component of position (accuracy decreased with position; *b* = −0.46, *SE* = 0.11, 95% CrI [−0.68, −0.25]) with no signs of an interaction (see Fig. 1, Panel A). Second, we employed conditional accuracy functions (Fig. 1, Panel B), which describe how the response accuracy for a given condition varies across response speed. This allows us to examine whether the effect of bigram frequency occurred across the entire range of RTs or was limited to early or late responses. As shown in Fig. 1 (Panel B), the conditional accuracy functions are approximately similar in the two conditions: regardless of speed response, responses were less accurate for the transposition of frequent bigrams than infrequent bigrams. Furthermore, both functions followed an inverted U, where the fastest and, to a lesser degree, the slowest responses were less precise.

## Discussion

The present study examined whether a key marker of orthographic processing, position invariance, emerges quickly after the repeated exposition of orthographic regularities—bigrams—in a novel script. To that end, we designed an experiment that followed, in its first phase, the same procedure as Chetail (2017, Experiment 1b). For around 9 minutes, participants repeatedly received a series of five-artificial-letter strings that contained a frequent bigram. Then, in a wordlikeness task, participants judged the strings with a trained bigram as more wordlike, thus showing that readers picked up subtle new orthographic regularities very rapidly (i.e., frequent bigrams in a specific position), replicating Chetail (2017).

In the second—novel—phase of our experiment, participants performed a same–different matching task in which they had to decide whether pairs of five artificial letter strings were the same or not. The critical test was whether the pairs involving the transposition of the letters of a frequent bigram (**AB**UVX—BAUVX) had a boost in confusability (i.e., position invariance in addition to position uncertainty) than those pairs involving the transposition of an infrequent bigram (**XZFG**U—ZX**FG**U) (i.e., position uncertainty). Responses to pairs with a letter transposition in a frequent bigram were less accurate than those with a letter transposition in an infrequent bigram (54.7 vs. 62.0%, respectively), thus showing an increase in confusability—the latency data were in the same direction.^3^ Critically, this is the first demonstration of the rapid emergence of position invariance in a novel script with an experimental design (see Rumelhart, 1985, for comparable evidence with legal vs. illegal bigrams in English).

Altogether, these findings favor the view that print exposure alone facilitates the development of orthographic regularities (see Chetail, 2017; Chetail & Sauval, 2022). Critically, the repeated exposure to patterns of letter co-occurrences would facilitate the development of internal representations of letter clusters (i.e., frequent bigrams), inducing position invariance (seeGrainger & Ziegler, 2011, for a model of word recognition where chunking frequent letter combinations plays a critical role). As a result, when individuals are presented with BAUVX, the cognitive system would often confuse it with **AB**UVX because (1) BA has not occurred before and **AB** has a precise mental representation, and (2) there is positional noise during order assignment. In contrast, only perceptual noise would affect order position in the strings that involved infrequent bigrams, such as XZ**FG**U and ZX**FG**U. Thus, the combination of these mechanisms can readily explain why BAUVX is more confusable with **AB**UVX than XZ**FG**U is with **ZXFG**U. A parallel rationalization also applies to the confusability of praikc with praick (Rumelhart, 1985).^4^

Our findings also shed light on the early developmental trajectory of orthographic processing when learning to read. Firstly, statistical learning would facilitate acquiring orthographic regularities, such as frequently co-occurring letter combinations, that support the subsequent processing of higher-level linguistic entities. This idea fits well with the fact that preschoolers become rapidly sensitive to bigram frequency because of its functionality in learning to read (Mano & Kloos, 2018). Secondly, the extra confusability of transposed-letter pairs of frequent bigrams suggests that, in the first moments of learning to read, position-invariant processing is tuned to the processing of frequent letter chunks, helping the subsequent encoding of words. Repeated exposure to a bigram (e.g., **AB**) clues us in that it is probably present in many new to-be-learned words; hence, position-invariant processing is adjusted to encode **AB** for both **AB** and BA, with an optimization purpose. Thus, orthographic learning would involve optimizing the mapping of letter-level information onto higher-level representations.^5^

To sum up, the present findings demonstrated that orthographic regularities in the form of bigrams help the rapid emergence of position invariance when exposed to a new script. Importantly, this mechanism has an adaptive purpose: to help encode the to-be-learned words. In short, the ability to encode the properties of sublexical orthography may represent a unique ability within reading development, thus opening a window to further experiments examining sensitivity to orthographic regularities in early childhood.

All the raw data and analyses are available online (https://osf.io/nst8w/?view_only=99edab01958a4d7cb8e8dc0c44d001ea).

## Figures and Tables

**Fig. 2:**
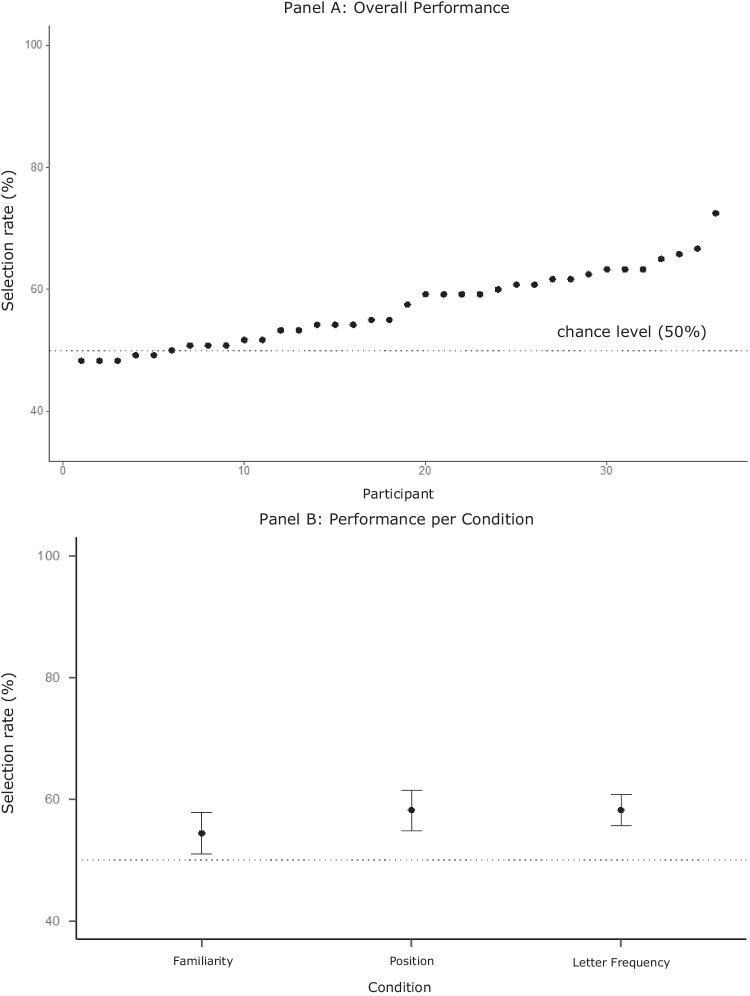
Panel **A:** Individual performance in the wordlikeness task. Panel **B:** Selection rate of critical items in the familiarity, position, and letter frequency conditions

## Appendix A

### Analyses of Phase 1

For the inferential analyses of Phase 1, we reproduced the analyses of Chetail (2017)and employed generalized linear mixed-effects (GLME) models in R (R Core Team, 2021) using the lme4 (Version 1.1-27.1) package (seeBates et al., 2015) and the lmerTest package (Kuznetsova et al., 2017). The dependent variable was accuracy, and we fitted GLME models with no fixed factor (comparison with chance level in the wordlikeness task: glmer(ACCURACY~1+(1|PARTICIPANT)). As a short teaser, we essentially replicated the findings.

**Exposure:** The detection rate of fillers was 99.74%.

**Wordlikeness**: Overall, participants performed above chance level (M = 56.94%, see Fig. 2, Panel A), b = 0.28, SE = 0.04, *z* = 6.71, *p* < .001. The performance was significantly higher than chance level in the familiarity condition (*M* = 54.44%), *b* = 0.18, *SE* = 0.06, *z* = 2.63, *p* = .008, in the position condition (*M* = 58.19%) , *b* = 0.34, *SE* = 0.07, z = 4.83, *p* < .001, and in the letter frequency condition (*M* = 58.19%), *b* = 0.33, *SE* = 0.05, *z* = 6.19, *p* < .001 (Figure 2, Panel B). Furthermore, performance was significantly higher for the initial bigram than for the final one (*b* = 0.24, *SE* = 0.08, *= 2.83,* *p* = .005).

## Appendix B

Results of replaced-letter trials and *same* trials in the same–different task

*Replaced-letter trials.* Responses were only 3 ms faster for the frequent bigram replacements than for the infrequent bigram replacements (613 vs. 615 ms; *b* = 0.00, *SE* = 0.02, 95% CrI [−0.03, 0.04]). Moreover, there were no signs of differences in accuracy for the pairs with a replacement of frequent bigrams versus infrequent bigrams (76.59 vs. 78.88, respectively; *b* = 0.14 *SE* = 0.12, 95% CrI [−0.10, 0.37]).

Same trials. There were virtually no differences in response times between pairs with frequent and infrequent bigrams (603 vs. 608 ms; *b* = 0.01, *SE* = 0.01, 95% CrI [−0.02, 0.03]). Regarding accuracy, accuracy was higher for the pairs containing a frequent bigram than for the pairs containing an infrequent bigram (89.72 vs. 87.55; *b* = −0.33, *SE*= 0.14, 95% CrI [−0.62, −0.06]).

## Footnotes

1 Bold and underlining indicated, in the examples, the frequent bigram and the letter manipulation, respectively (see Table 1 for examples of the employed stimuli).

2 Bayes factors (BFs) were computed with JZS priors using bfrms (Singmann & Gronau, 2021) in R (R Core Team, 2021). BFs above of 3, 10, and 100 represent moderate, strong, and conclusive evidence, respectively.

3 Transposition effects in the same-different matching task are typically more robust for accuracy than for RTs (e.g., Duñabeitia et al., 2012; Fernández-López et al., 2021; Massol et al., 2013). This is not surprising when considering that the relatively high number of errors for transposed-letter pairs makes the latency data more variable and noisy than in paradigms with close-to-ceiling performance.

4 Notably, although the orthographic regularities modulated the encoding of the order of letters, they did not have an effect in the encoding of letter identity: replacing a frequent or an infrequent bigram produced similar results.

5 One might argue that the locus of position invariance in the present experiment occurred at the letter level. The idea is that participants could have learned extremely position-specific representations of letters rather than letter co-occurrences: Instead of learning that the letters **AB** co-occur, participants could have learned that A always appears in the first position of the string and B in the second. While this is a plausible interpretation, we should note that more than one frequent letter could be placed in the same position (e.g., the second position can be occupied by a B (from AB___) or a C (from _CE__). Thus, there was not a one-to-one relation between letter and position, which makes the interpretation of learning position-specific representations unlikely (see Cassar & Treiman, 1997; Pacton et al., 2001; Rothe et al., 2015, for research showing that children are sensitive to the position of letter co-occurrences rather than the position of single letters). Furthermore, the view that a newly learned letter could be linked to a specific position might work for external letters—which would act as anchors—but not internal letters; in the current experiment, the transposition letter effects were similar in size for all positions. We acknowledge, however, that further research should be conducted to test this possibility.
